# When Peppa Pig and Confucius meet, joining forces on the battlefield of health literacy–a qualitative analysis of COVID-19 educational materials for children and adolescents from China, the USA, and Europe

**DOI:** 10.1371/journal.pone.0278554

**Published:** 2022-12-06

**Authors:** Maria Świątkiewicz-Mośny, Anna Prokop-Dorner, Magdalena Ślusarczyk, Natalia Ożegalska-Łukasik, Aleksandra Piłat-Kobla, Joanna Zając, Malgorzata M. Bala

**Affiliations:** 1 Institute of Sociology, Jagiellonian University, Kraków, Poland; 2 Department of Medical Sociology, Jagiellonian University Medical College, Kraków, Poland; 3 Faculty of International and Political Studies, Jagiellonian University, Kraków, Poland; 4 Department of Hygiene and Dietetics, Jagiellonian University Medical College, Kraków, Poland; Nanyang Technological University, SINGAPORE

## Abstract

In times of pandemic, health literacy (HL) is very important, as it helps to find, understand, and use essential health information and services. According to WHO, HL is pivotal in fighting infodemic effectively, and education is a vital tool for developing it. In the presented work, we analyze 247 educational materials dedicated to children, adolescents, and their carers explaining the pandemic, prepared by the Chinese, American, German, Italian and Polish governments and international non-governmental organizations. Focusing on the textual and visual side of the documents, we investigated how the pandemic is explained and what discursive measures were used to inform young citizens about the risks and consequences of pandemic restrictions. Additionally, we verified whether the materials helped developing critical thinking, which is crucial to prevent spreading fake news and conspiracy theories. Although the analyzed materials were prepared in different cultural contexts, we identified that all of them contained simple instructions on the desired behaviours during the pandemic. Key messages relating to the importance of hygienic behaviors were often supplemented with guidelines on how to successfully complete each action. While the cultural particularities in presenting the state of the pandemic are visible, the challenges of dealing with the emotional and social crises were dominant all around the world. In our study, we argue that the possibilities of building HL were not fully exploited by the national and international institutions. Citizens were taught how to behave in unusual circumstances but not why they should behave differently. The educational materials lacked reliable knowledge that would allow them to deal with infodemic and develop critical thinking. We conclude that health education expertise worldwide should be focused on enhancing individuals’ ability to make informed health decisions and provide three recommendations regarding the process of development of health educational resources for children and the youth.

## 1. Introduction

From the very beginning, the COVID-19 pandemic was accompanied by a wave of information that included alarmist reports, rapidly changing numbers, and (often contradictory) recommendations. A sanitary regime was imposed in most countries, and the WHO and national governments began to communicate with the public about the challenges the pandemic presented, urging the citizenry to change their lifestyles and to adopt the relevant sanitary requirements. Paakardi and Okan recommends that “*most valuable information is created in an easy-to-understand manner that offers simple and practical solutions*” [1] but unfortunately, while the management of the health crisis depended on citizens’ conformity with the sanitary restrictions, the media, and in particular social media, have demonstrated their vulnerability to what is known as fake news. Two decades ago, Eysenbach [2] coined the term “*infodemic*” to describe this phenomenon. The WHO adopted the word in a report on 2 February 2020 to describe “*an over-abundance of information–some accurate and some not–that makes it hard for people to find trustworthy sources and reliable guidance when they need it when massive misinformation and conspiracy theories relating to COVID-19 were circulating widely on the internet”*. The WHO and the UN have since sought to combat this infodemic; however, the fight against fake news resembles King Canute’s order that the tides cease.

On an individual level, one resource that helps in the fight against both the pandemic and the infodemic is health literacy (HL), a term derived from the word literacy as opposed to illiteracy, i.e. those basic skills necessary for us to read texts and understand them properly. HL is closely related to health behaviours and decision making in all age groups [3, 4] and in some countries (e.g. the USA, Canada, and Australia), it is also treated as a national health indicator [5]. HL is also one of the four pillars of infodemic management proposed by Eysenbach [2]: first, information monitoring, second, building e-HL (or DHL: digital health literacy), and science literacy capacity; third, checking and monitoring facts; and, fourthly, ensuring that knowledge is conveyed in such a way as to make the message as unsusceptible to distortion as possible [6–8].

While an increasing number of countries appreciate the importance of HL and seek to measure its level [cf. 5], an important question remains concerning how to *increase* its level. Mei et al. [5] highlight the fact that while knowledge is easily and quickly acquired, the formation of beliefs and the subsequent production of health-related behaviour takes longer and requires numerous efforts in the area of education and health promotion. In April 2020 (just one month after the WHO declared COVID-19 to be a pandemic), Paakardi and Okan [1] commented in the Lancet claiming that HL is still an underappreciated competence. This was particularly important at a time when countries all over the world were facing a rapid increase in deaths due to COVID-19, and the international community did not appear to have the tools to change this. They also stated that *“the development of health literacy is even more topical than ever to prepare individuals for situations that require rapid reaction”* [1]. Low HL levels have been diagnosed and described as a problem [5], and various measures have been taken to address the situation; however, the discussion about health education, HL, and DHL has only intensified since the start of the pandemic. Increasing numbers of studies have suggested making HL and DHL mandatory topics in school and university curricula. This corresponds to the recommendations made by the OECD in the PISA report “21st‑Century Readers. Developing Literacy Skills in a Digital World” [9], which indicated the necessity of developing literacy skills and especially digital literacy competencies in view of the growing complexity of the modern world and the increasing presence of digital technologies in our lives, especially in periods such as that of the COVID-19 pandemic.

In this article we aim to investigate how the COVID-19 pandemic is presented to the youngest generation and how various national and international actors, as well as non-governmental organizations from different cultural contexts communicated the COVID-19 pandemic to children and young people. Furthermore, we focus on answering the question of whether materials prepared for children and adolescents during the pandemic have the potential do build HL. Our objectives included: 1) exploring how the pandemic is explained to children and adolescents and which topics related to the global health crisis were present or absent in these educational materials; 2) analyzing what discursive means were used to inform young citizens about the threats and the consequences of the restrictions brought in because of the pandemic; and 3) establishing whether the materials cover critical thinking, as in the face of the spread of fake news and conspiracy theories, this competence is key in navigating the new challenges brought by the pandemic situation. This study will form the basis for recommendations for development of the health educational resources.

## 2. Materials and methods

Following Berkman et al [10] we define HL as “the degree to which individuals can obtain, process, understand, and communicate about health-related information needed to make informed health decisions” [p. 16; see also 11–13]. Consequently, HL relates to a variety of skills, from the ability to communicate with specialists who provide knowledge about health, to the competences of searching for information about the impact of various factors on health and one’s critical ability to evaluate this information. Zarcadoolas et al [14] highlights the following dimensions of HL: 1) fundamental literacy (essential competencies and skills enabling individuals to access the necessary knowledge about health), 2) science literacy (knowledge about basic scientific and technological concepts, but also an awareness of the uncertainty present in even well-grounded theories), 3) civic literacy (a conscious and critical reception of messages presented by the media and the rational application of information), and 4) cultural literacy (the competence to understand collective beliefs, habits, stereotypes, etc. in the process of interpreting and using health information). This means that social competences such as communication skills and critical thinking (CT) are prerequisites for taking appropriate decisions about health in daily situations [15].

In response to the global health crisis in 2020, many national governments, international organizations, and non-governmental organizations, as well as private entities and individuals, undertook efforts to educate communities facing the anomie that resulted from the pandemic’s disruption of social norms [16]. We conceptualized the educational materials developed and disseminated by public institutions as a part of the educational discourse on health. We applied a qualitative method of discourse analysis [17] to describe discursive means used by governmental institutions and national and international agencies in the USA, Germany, China, Italy, and Poland, as well as across the international community (e.g. WHO, PAHO, UNICEF) to convey the message on the outbreak of coronavirus and the new order of daily life routines to the recipients: children and young people. The analysis included studying educational material content, discursive means (e.g. linguistic, visual, editorial) and narrative strategies (e.g. plots, protagonists, settings) used.

The countries included in this study were selected according to two criteria: the dynamics of the pandemic, and the educational capital. We operationalized the former in regard to pandemic time span. We selected five countries hit by the pandemic crisis in different points in time and that fought the spread of the virus through various levels of control measures, faced varied levels of incidence and mortality rate, and approached the pandemic against a background of different healthcare system organizations (S1 File). We assumed that the latter criterion, operationalized as national mean scores in the Programme for the International Student Assessment (PISA), indicated to what extent education has been prioritized in each country. We compared the PISA national mean scores of the key skills of 15-year-olds (reading, mathematics, and science) and their digital literacy. Student performance in these areas is crucial in gathering knowledge, engaging with and participating in society, making well-founded judgments, and drawing evidence-based conclusions about science-related issues [9].

We used search engines like Google, Bing, Duckduckgo, Yahoo, as well as Baidu, Sohu for Chinese materials and webpage of chosen organizations (like WHO, PAHO, Ministry of Health of each analysed country, all mentioned in the Table 1 to identify educational materials dedicated to children, teenagers, and their parents or teachers, and that covered such topics as: COVID-19, the pandemic, the consequences of a pandemic, and COVID-19 vaccinations. Moreover, we searched through the websites and social media of national and regional agencies from five countries and of global and regional organizations—the WHO, PAHO, UNICEF, UNESCO, World Bank, ICRC, SAFE kids worldwide, IBCR, Save the children, PLAN international, World Vision, Aflatoun, the European Commission, and the Council of Europe. The search process was conducted by researchers with cultural and linguistic competences relevant to the analyzed country. Next, the preliminarily selection of materials was discussed by the research team and arising doubts regarding inclusion resolved.

**Table 1:** Educational materials about the pandemic designed for children, adolescents published by American, Chinese, Italian, German, and Polish national institutions and international organizations included in the analysis.

INTERNATIONAL ORGANIZATIONS	USA	CHINA	EUROPE		
			ITALY	GERMANY	POLAND
21 textual materials	51 textual materials	35 textual materials	10 textual materials	40 textual materials	10 textual materials
31 videos	12 videos	14 videos	7 videos	14 videos	2 videos
**Key content producers**					
World Health Organization The Pan American Health Organization UNICEF	Centers for Disease Control and Prevention, engage.youth.gov	National Health Commission of the PRC Ministry of Education of the People’s Republic of China, Chinese Center for Disease Control and Prevention, educational/ public institutions (all with approval from Chinese Communist Party)	Ministero della Salute, Save the children Italia, hospitals, educational institutions, Ufficio federale della sanità pubblica	Die Bundeszentrale für gesundheitliche Aufklärung. Bundesamt für Bevölkerungsschutz und Katastrophenhilfe, Bundesministerium für Bildung und Forschung	Ministerstwo Edukacji, Ministerstwo Zdrowia
**Availability of educational components enhancing critical thinking**					
PARTLY	YES	NO	NO	YES	NO
				• the possibility to ask questions emphasizing (in parenting materials) the importance of creating a space for questions and discussion • references to the infodemic, over-reporting	
Only in materials for parents, teachers, and adolescents, not for children • some references to infodemic, misinformation, and believing reliable sources	• the possibility to leave comments under a post • references to infodemic and fake news	• a single material pointing out the problem of the reliability of sources			
**Racial, cultural and gender diversity**					
PARTLY,	YES,	YES	PARTLY,	YES,	NO
• some materials presented a racial, cultural or gender diversity of characters	• racial diversity of people presented in most materials (visual context) • cultural diversity	• racial diversity of the characters presented in most materials (visual context) • visual elements specific to Chinese culture (Spring Festival, decorations, architecture)	• in some materials mentioned but in a manner avoiding the apportioning of blame for the pandemic	• racial diversity of the people presented in most materials (visual context) • language (materials prepared for migrant communities, including different language versions–translations available in 31 languages)	
**Language of the materials and available translations**					
English and Spanish (PAHO) and French, Arabic, Russian, Chinese versions available for some materials	Official national language (English), a few materials available in Spanish	Only the official national language (Chinese), a few materials available also in English	Only the official national language (Italian)	Official national language (German), one material translated to 31 languages	Only the official national language (Polish)

Next, the materials were coded by six trained coders. To start with, we performed an inductive coding to develop a coding book, which was tested and further enhanced during discussions within the coding team. Once the codebook was established (S2 File), the material was coded deductively. The coders’ reliability rate was calculated with Kappa (RK) and came to 0.83. The codebook covered 13 main codes, divided into 96 sub-codes, which helped with organizing the information covered in the educational materials. The codebook also covered the narrative approaches used in conveying the educational message (i.e. the structure of the narrative, the type of language used, and the type of knowledge presented). Attention was also paid to the nomenclature and metaphors applied to describe the virus and the situation resulting from its spread. Additionally, for each piece of educational material we explored which new social practices were discussed and whether critical thinking was promoted. Finally, we analyzed to what extent socio-cultural diversity, including gender and ethnicity was reflected in the materials.

Supported by the analytical software MAXQDA, we analyzed the textual and visual material in the light of our research questions. We identified the central notions in the materials produced by each group of authors (according to country and international organization). Case summaries were written (Table 2) to characterize the dominant patterns in the documents from each country and visual tools were used to compare the sets of materials between the groups of authors. Those patterns that emerged were discussed further by theme. Reporting of the study has been prepared in accordance to the Standards for Reporting Qualitative Research (SRQR).

**Table 2:** International and national educational materials on COVID-19 pandemics - case summaries.

INTERNATIONAL ORGANIZATION	USA	CHINA	EUROPE		
			ITALY	GERMANY	POLAND
**Key prevention means:** washing hands, disinfection, masks, coughing hygiene, how the virus spread and how to stop spread, avoiding contacts, physical distance, mental health, social health, follow local authorities To children: mainly washing hands, cough etiquette, **Key discursive means used:** • using well-known cartoon characters to encourage safe behaviours • main message: stop spreading • Directive instructions: what to do/what not to do; keeping daily routine	**Key prevention means:** wearing mask correctly, social distance, handwashing, caring for mental health (coping with stress, talking about emotional consequences of pandemic, mental health crises), alternative strategies for organizing rituals, celebrations (Christmas, Halloween), daily challenges in pandemic (parenting, cooking, media exposure, leisure) Less common prevention means: cleaning the closes surrounding, contact tracing, increased testing. **Key discursive means used:** • redirecting to further sources • colloquial language	**Key prevention means:** wearing masks, social distance disinfection of everything, opening windows, washing hands often, avoiding public places, physical exercises, eating regularly (not eating raw food and wild animals meat), hygiene (preventing coughing, sneezing) Almost no remarks about caring for mental health (just in few materials) **Key discursive means used:** • the language often is very emotional, bombastic, • references to “heroes” and “warriors” • plays with words like “scary” and “afraid of”, which are always followed by the expression of assurance that “we will survive and make everything ok again TOGETHER” • very often remarks to how COVID-19 started; that human crossed the border of wild animal life, remarks to ecology, balance, necessity to stop eating meat of wild animals • history of coronavirus (coronavirus has been existing before) • political context (exemplary for the world)	**Key prevention means:** washing hands, wearing masks, avoiding contact with grandparents, staying at home (why children cannot play outside), how to greet at school, which daily behaviors now are dangerous (e.g. ways of sharing food) **Key discursive means used:** • the way of presentation is calm, not scary, optimistic • most of the written materials are designed for adults to support them with ideas how to spend time at home	**Key prevention means:** wearing mask correctly, social distance, handwashing; mental and social health (coping with stress, talking about emotional consequences of pandemic, support strategies); daily challenges (distance, school, lack of contacts); support for parents (daily challenges, mental health, advice); practical materials for kindergartens/ schools (posters about handwashing etc.), keeping a daily routine **Key discursive means used:** • instructional language, focusing on what is within our power to control and what is safe for us • care not to scare, references to songs, warm stories with a sense of humour, use of fairy tale heroes or new characters close to the children (a friend, a mascot/teddy bear, a superhero)	**Key prevention means:** hands washing, wearing masks, other means (avoiding Corona-parties, safe sneezing); how SARS-CoV-2 spreads; consequences of isolation: mental and social health (coping with emotions, organizing time in a new manner and online learning/ teaching; challenges connected to e-learning; psychological support for pupils/parents/ teachers **Key discursive means used:** • most of the materials are intended for adults so that they remind children about safety rules (wearing masks, distance). • using directives: what to do / what not to do • information provided mainly in the form of posters and / or infographics

## 3. Results

Out of initially identified 295 educational materials, eventually 247 met our inclusion criteria. Textual materials (170) dominated over videos (77). In the former category there were mostly very short and short formats such as short brochures, social media posts, checklists or posters. We identified 16 books and extended articles. In the latter category there were short films, animations, interviews as well as songs dedicated to the pandemic.

The analysis enabled us to identify some commonalities in the content and means used to explain the pandemic to children and adolescents. Some notions, such as washing hands, social distancing, and wearing masks, as well as taking care of one’s psychological well-being, were promoted in all five countries as well as by the international organizations. However, each country has its own particularity regarding the cultural context, public health policy, and the dynamics of the pandemic, with these being reflected in the educational materials designed for the youngest generation (Table 1).

### 3.1. Naming the pandemic situation–does the virus have its crown?

First, the most elementary difference is related to the naming convention. From a scientific perspective, SARS-Cov-2 is the official name of the virus, and COVID-19 is the disease (or the set of symptoms) caused by that virus. In everyday use, the word "coronavirus" appeared predominantly to describe this new, highly infectious virus. However, in official documents and educational materials, especially those prepared by international organizations and in the United States, the term ‘COVID-19’ was used most often. Some minor deviations to these general observations were identified, such as that the Italians seem to lean towards the term "virus," while at the same time “germs” is sometimes used in the materials produced in the United States, and the name “coronavirus” was seldom shortened to “corona” in the German resources. It seems that when it comes to naming the virus and the disease it causes, only China stood out. In the materials from China analyzed, the most commonly used term was "new coronavirus" or, less commonly "new type of pneumonia". The less common use of the abbreviations COVID-19 or SARS-CoV-2 is perhaps related to the fact that China, unlike the other countries analyzed, had the greatest experience with the first SARS-CoV epidemic in 2002–2003, so to emphasize the fact that the infections were caused by the new virus, the expression "novel coronavirus" might have been used intentionally. Very often virus is called as “a little monster”.

The period of the pandemic was presented in the various countries differently. In China, it was described as an absolutely unique time, because the lockdown coincided with the celebration of the Chinese New Year, which in Chinese culture is a time of national migration, with much of the populace moving from one place to another to celebrate with their family. The Chinese children’s materials are imbued with the metaphor of war and struggle, of something that everyone must fight through together. And although this time is full of challenges, and the experience of the lockdown is extremely difficult, this fight will certainly be won, thanks to collective cooperation (S3 File). In the German and American materials, this idea that the period of the pandemic is a time full of challenges was also emphasized, and at the same time working together was encouraged.

Difficult questions are not avoided, such as whether children can visit their grandparents or meet their friends, and parents are also encouraged to assert control over the media used by their children so as not to increase their level of stress with the situation. In the Polish materials, in the context of describing the period of the pandemic, there is clear signalling that the pandemic will be brought under control within an unspecified period of time, but that the effects of the pandemic will certainly be felt for a long time in all areas of life. The Italian materials avoid describing the period of the pandemic as a difficult time by using just the terms “isolation”, “lockdown”, and “quarantine”. Mostly these materials sought to highlight the importance of obeying the new, temporary rules for the safety of loved ones and for everybody in society. Quite surprisingly the motif of the loss of loved ones occurred (e.g. the death of one’s grandparents), probably due to the high mortality rate in Italy where focussing on this issue appeared advisable. The materials used in the United States describe the period of the pandemic as one of uncertainty and enormous mental stress. They also underline the fact that periods of social isolation might result in the breakdown of mental health. The American resources also emphasize the fact that there are ways to solve these problems, primarily by using professional help. A more detailed explanation of the source of the pandemic is unique to the Chinese narration. This follows the most common justification—provided, among others, by the WHO and the Chinese government—that the source of the SARS-Cov-2 pandemic can be traced to wild bats. It is stressed that SARS-Cov-2 is a novel member of the wider coronavirus group, which is known to science and has been studied for many years. In the educational materials analyzed, we observed slight differences resulting from the ways in which the concept of the virus was used. However, the iconic representation of the virus is very similar in all of the materials analyzed: the virus is commonly presented as a sphere with protrusions forming a crown (S3 File).

Following the theory of the social construction of reality [18] and assuming the importance of language in the description of reality, the way the pandemic is presented in the educational materials is highly important. This not only builds a narrative, but above all mobilizes (or fails to mobilize) the resources necessary to deal with the situation. It seems of particular importance whether words are used to construct an atmosphere of danger and anxiety, or rather to motivate to action. The general aim of educational materials is the development of pupils, but in this context, it is also to introduce a change in social practice towards efforts appropriate to handling the pandemic.

### 3.2. Discursive means: Metaphors, heroes, and types of knowledge

While the linguistic means applied in the materials designed for children differ, it is the regional disparities that are most interesting. The vast majority of the materials analyzed presents lay knowledge, based on a common experience, non-professional terminology and not requiring specialised knowledge to understand issues linked to health, except for the content prepared by international organizations. This mainly comprises expert knowledge written in scientific language. Professional content also appears among selected Chinese materials meant for parents. However, in this case, it is merely the appearance of the figure of an expert, used as an authority in the field, rather than a way of communicating knowledge in a scientific and detailed manner. Lay knowledge predominates in the materials from the European countries. The instructional form dominates in the materials from all of the countries analyzed. The language used therein is directive, and the content is frequently presented in bullet form, more like a presentation than an essay. However, it is immediately apparent that the Chinese materials for children take more of a distinctive storytelling form. Short stories are presented, about how the pandemic started, why lockdown was introduced, how people can catch the virus, about the symptoms of the infection, and how children can join the fight against the pandemic. The most common characters are the virus and a child, though sometimes a parent or caregiver is included. The child is usually expected to listen to the adult or understand their indisposition due to the pandemic and the spread of this novel virus (S3 File).

Some of the authors of the educational materials explain the reality of the pandemic using a small selection of memorable metaphors. In the Chinese documents, the metaphor of war and struggle and individual responsibility is clearly evident. The virus is portrayed as a little monster that can be defeated only by fighting together (S3 File). Occasionally the German materials also use the metaphor of a mission to battle the virus, portrayed as a monster determined to defeat humans (S3 File). In the face of the threat of the pandemic, children are portrayed as important players. In the materials promoted by international organizations, such as PAHO or UNICEF, younger readers are empowered by the courage of the heroes to battle “the wicked virus” (S3 File). Through their adoption of a common strategy, children are able to participate in the fight against the pandemic. In the booklet from “My hero is you: How kids can fight COVID-19" a small girl learns that “There are many heroes keeping people safe from the coronavirus, like wonderful doctors and nurses. But you remind me that we can all be heroes, every day, and my biggest hero is you” (S3 File). A similar metaphor is used in an American video educating children on how to stay safe during summer camp: “Campers, get ready to stop COVID-19 in its tracks!” It reformulates following the rules of the pandemic into scouts’ honorary degrees: Peer protector, Germinator Terminator, Social Distancing Star, Face Masked Marvel, and Clean Camp Champ. Campers, counsellors and staff are encouraged to make the community healthier and safer by staying home when sick, washing hands for 20 seconds after touching things that other people use, keeping their distance when possible, and wearing a mask (S3 File).

A fascinating notion specific to Chinese culture was also observed. The figure of an ancient sage–presumably Confucius–appeared in one of the materials (S3 File). He demonstrated—together with his apprentice—the proper routine for washing hands. It is an example of how the authority of a figure like the Chinese philosopher and politician, whose influence on Chinese philosophy and education has been enormous for hundreds of years, might be used. As mentioned earlier, the Chinese materials do not avoid referring to the source of the pandemic. This is also present in the symbolic layer. The cause of the appearance of the virus is attributed to a "bad" human eating a wild animal (with the symbol of bat being used to exemplify the animal). The entire narrative is a warning against eating the meat of "unusual animals,” but beyond that the theme is further exploited, becoming a reason to discuss the issues of ecology and the ecosystem (S3 File).

In German, metaphors were mainly used in reference to cooperation, firstly to defeat the virus, secondly to protect medical staff, and finally to emphasize that we need to work in solidarity across the generations, not against each other. An empathic attitude towards those at risk of experiencing more serious COVID-19 outcomes was also promoted in some of the materials from the USA:

*Do them all to best protect yourself*, *your family*, *your friends*, *and those more likely to get very sick from #COVID19*. (S3 File)

Regarding representations of different genders in the materials analyzed, there appears to have been some effort to maintain a balance, albeit with a slightly greater number of male characters observable in both the Chinese and German cases. We found that the "hero" who engages in the fight against the pandemic fight was more likely to be male in the Chinese materials. In the German case, in those materials in which characters were explaining, advising or saving the world, male characters (humans or animals, e.g. special agent, doctor, singer) predominate. The stereotypical division of roles can also be observed, with a woman taking care of children and running the house (possibly trying to balance such responsibilities with work of their own) and with fathers fighting against the virus on the front line.

In the visual layer, icons of prohibition provide visual support for the restrictions and precautions discussed in the material. This iconography is consistent with omnipresent icons, such us the virus, a faucet (or tap) with water, soap, washing hands, maintaining social distance, masks, safely coughing and sneezing, children, children with adults, stress, and talking. The sound layer in the videos, in general, was intended to contribute towards a positive mood. Only in a few cases was the music fearful or threatening. In the Chinese video sequences, a kind of "revolutionary" melody was used, though only seldomly.

In terms of the structure of the materials, most of them are self-contained. The concept of linking was dominant in the case of the American resources, where all materials link to others, with the additional encouragement to broaden one’s knowledge (“*If you want to know more*…*”*).

### 3.3. A world focused on solutions

The educational materials tend to describe the pandemic as a potentially difficult time. On the one hand, this difficulty results from the overwhelming risk and level of uncertainty, together with the fear of illness and death; and on the other hand, from the imposition of sanitary regimes (washing hands, disinfection, social distance, wearing masks, staying at home). In educational materials, difficulties are presented as challenges and tasks that must be performed. This applies to caring for physical, mental, and social health. The materials are prepared in such a way as to avoid scaring children and adolescents. While the dramatic images of convoys carrying coffins in Italy that circulated in the media will remain a symbol of the pandemic (we refer here to the viral picture that turned out to be fake, https://www.reuters.com/article/uk-factcheck-italy-coffins-idUSKBN21F0XL), the educational materials do not mention any epidemiological data at all, and nor do they tend to deal with the problem of sickness or death. Only those prepared for Italian children raised the issue of grieving the death of loved ones and proposed solutions to help the children deal with their grief (S3 File).

The educational materials analyzed build a world focused on solutions. Most of the materials from the different countries outline potential problems that may result from the pandemic restrictions and offer instructions on how to face them. Some differences in the frequency of occurrence of some recommendations can be observed, which might imply the relative significance given to these recommendations in different cultural contexts. Washing hands appears to have become a key task during the pandemic and is presented most often in the educational materials. The materials are not concerned with "ordinary" hand washing but with the professionalization of the task. Hands should be washed in accordance with a set of instructions previously only followed at hospitals. Warm water and soap are required, as in the more ‘traditional’ approach to hand washing, but specific details are given as to when and for how long the hands should be washed. In the educational materials for children, the appropriate duration of the procedure is measured in terms of popular songs (e.g. “Happy Birthday” in the USA, special songs offered to children in Italy (S3 File) and in materials for adolescents as lasting for precisely 20 seconds. In the Chinese materials, very detailed instructions describing a full seven steps are presented so that the reader is informed of the correct hand washing procedure (S3 File). The German pupils receive even more intensive instructions on the correct hand washing procedure (instructions appear in 31 materials out of 55). Some of the materials, beside promoting specific behaviours, offer an explanation as to why they are so crucial. One of the Polish video materials explains that “many surfaces can be contaminated with the virus" (S3 File).

Videos with popular characters from cartoons, including Peppa Pig, Sesame Street were prepared by PAHO (S3 File). Washing hands is presented here as an enjoyable activity, with this aspect of enjoyment underlined by music that builds a positive mood, rather than representing the task as a chore that must be completed because of the pandemic.

While washing hands and wearing masks appear in many of the documents, the need for disinfection is not mentioned very often. While the American and Chinese materials designed for parents and teachers covered instructions on how to disinfect toys and how to ventilate rooms, the European ones did not. Materials prepared by international organizations mainly covered prophylactic actions such as washing hands, cough and sneeze etiquette, avoiding touching the face, staying home when sick, not shaking hands, maintain social distance, wearing masks, cleaning and decontaminating surfaces, cancelling community/school events, proper ventilation, and avoiding crowded places and contact with other people. The messages aimed at children appear mostly to focus on hand and respiratory hygiene.

Wearing masks is another new rule to follow in the world of the pandemic. The Chinese offer very intensive education on this point, with 21 out of 35 materials mentioning the necessity of wearing masks (S3 File). Children and adolescents from other countries also receive information about how masks are important. German audiences are instructed on how to wear masks properly (S3 File). The young audiences in Poland and Italy received similar instructions. The American materials informed their audience that wearing two masks is even better, with encouragement to use washable masks; instructions on how these should be washed were also presented (S3 File).

Another new rule that aimed at regulating social life and counteracting the impact of the pandemic was avoiding social contact and keeping one’s distance. This principle was particularly difficult to implement. On the one hand, it is “unnormal” to keep your distance in places such as schools and kindergartens and on public transport. Children play together, and adolescents generally spend their times in groups. On the other hand, dealing with the pandemic required the suspension of traditional social contact. This led to a feeling of isolation and exclusion. Children were encouraged to use different media to keep in contact with their relatives (their grandparents, for instance) and/or friends (S3 File). As mentioned above, within the educational materials there is a lot of information about prophylaxis but hardly any discussed treatment. It is likely that this was connected to the fact that, at the time of the study, there was a paucity of positive messages to communicate. International organizations mentioned future immunization, and this theme appears in the American materials as well:

*Take care of your body*. *(…) Continue with routine preventive measures (such as vaccinations*, *cancer screenings*, *etc*.*) as recommended by your healthcare provider*. *Get vaccinated with a COVID-19 vaccine when available*. (S3 File)

The German government also taught pupils about vaccination. It should be mentioned that in Spring 2020 and Autumn 2020 there was no COVID-19 vaccine available. The German government, in introducing the subject of vaccination against COVID-19, thus began an educational and information campaign. Their materials suggested that a vaccine was in development, and stressed the fact that vaccine development is a long-term project. The material also indicated which groups would be vaccinated first.

*Why is the vaccine taking so long*? *Scientists are developing a vaccine so that people can be protected against the coronavirus*. *Actually*, *it takes quite a long time to develop a vaccine*. *Information about the virus has to be collected*. *At the same time*, *the vaccine has to be safe and has to be tested on a large number of people*. *It is especially important to exclude side effects*. (S3 File).

Vaccination was also present in international organizations’ educational materials with the message adjusted to four different audiences (S3 File).

Educational materials prepared by international organizations often listed the symptoms of COVID-19 (cough and high temperature). This theme was also present in the Chinese, Polish and Italian materials. Germany offered an even closer discussion of how the virus spreads (S3 File).

The educational materials also include information about who may be most strongly affected by the disease; however, these are marginal items. This is information that can convey uncertainty. It should also be remembered that when the materials were being prepared, detailed knowledge about how SARS-Cov2 worked was relatively scarce. Scientists were working on treatment and vaccination. In 2020, intensive research towards the search for a cure and a vaccine against the virus continued. Prophylaxis was the only tool available. The media became involved in the fight against the virus by providing data on the number of cases and deaths related to COVID-19 on a daily basis; however these numbers (of people infected and dying) do not appear in the materials. Even on social media channels whose core audience is young people (such as youth.gov), this information was not shared. The aim here was perhaps to avoid scaring children and adolescents with numbers that reflected the scale of the pandemic, and the seriousness of the situation was generally not built up through frightening statistics. The pedagogical play approach dominates, which indicates that through play it is possible to manage educational activities. Research in the area of neurodidactics has generally confirmed the effectiveness of this approach [cf. 19, 20]. However, the lack of educational activities that would help build knowledge and understanding of the epidemic situation using numbers, and that would teach the target audience how to deal with this type of information is somewhat puzzling.

Building health competences in educational materials focuses on encouraging pupils to take preventive measures. Much attention is paid to mastering specific behavioral patterns (washing hands, wearing masks, maintaining social distance), but little to developing knowledge. Knowledge, in turn, could be the tool that will help to critically evaluate the information received.

### 3.4. How should the challenges of the pandemic be dealt with?

The materials analyzed contain many tips on how to deal with the challenges of the pandemic. As mentioned above, all of the documents emphasized the importance of physical, mental, and social health, but the dominant topic seems to be mental well-being, encompassing the topics of coping with the stress caused by isolation, anxiety, depression, or, more generally, the rapidly changing living conditions: *“feelings of anxiety and panic*, *problems with sleep*, *and concentration of attention can lead to emotional and mental crises”* (S3 File). There are numerous specific instructions presented in the educational materials for children and adolescents. Some focus on daily routine: how to deal with isolation and/or online school; while others concentrate on special events like holidays.

American children and adolescents can find very precise instructions about healthy daily routines: eating well, working out (remaining physically active) and keeping in contact with friends should help in surviving this difficult time.

*To whoever needs to hear this*: *it’s ok to set boundaries around usual holiday traditions*, *travel*, *gatherings*, *people and situations generally* *t**o protect your mental and physical health*. *(…) Take Charge of Your Health Fitness Nutrition Physical activity is a great way to relieve stress—pandemic-related or not*. *Learn how to eat healthy*, *be physically active*, *and take charge of your health*. (S3 File)

The most vivid notion presented in the materials was the holistic approach to well-being: physical fitness, mental functioning, and maintaining satisfying connections with others.

American organizations also prepared very detailed instructions for parents and caregivers, providing ideas on how to adjust parental strategies to fit the new context. Elmo’s mum from Sesame Street shares her struggle with parents and advice talking about emotions with children (S3 File). Parents’ attention is drawn to providing their children with reassuring routines:

*Set up routines and schedules at home*, *and keep your child focused on these*. *Knowing what to expect at home will be comforting*. *Set timers and give regular reminders for upcoming transitions and before you change activities throughout the day*. (S3 File)

Parents in the USA also received the suggestion to limit access to media, as this may increase the stress and tension experienced by children. Various fun activities involving whole families that could be carried out at home were proposed. The authors suggested imagining a return to the norms of the 1990s:

*So*, *what CAN we do*? *Pretend it’s 1990*, *not 2020*. *Take walks and play outside*. *Cook or bake together*. *Have a family movie night*. *Have a family game night with charades*, *Pictionary*, *card games*, *or dust off those old board games or puzzles*. (S3 File)

Chinese educational materials likewise focus on the emotions and mental state that can be triggered by isolation and the pandemic (feelings of fear, panic, being in a small space 24 hours a day so close to your loved one). Chinese parents have their role—they have to provide a positive example, use the time in which they are with their children to do many things together (cooking, drawing, playing), but also allow the children to do their work.

In the educational materials produced for European pupils, the psychological consequences of isolation, such as psychological stress and mental problems, are also taken into account, but they do not seem to be the most important messages. The Italian materials for children focus on necessity of isolation and staying at home, not visiting grandparents and the curtailment of visits paid to friends, while those that focus on adolescents suggest full day schedules that can help to maintain a normal life (S3 File). In Poland children and adolescents can find the phone numbers of organizations that offer help and psychological support free of charge (S3 File). The German materials proposed some solutions like quizzes and tutorials for handicrafts, and suggested simply trying to wait out these difficult times. For parents, the materials offer many suggestions, from getting enough sleep and thinking about good nutrition, to tips for dealing with isolation and distance, to providing contact details to experts and professionals who can help.

*Try simply to be there for your child*. *Often it is already a great help if a familiar caregiver is nearby*. *A structured daily routine with fixed sleeping and eating times provides support and security*. *Make sure that familiar things are kept as much as possible*, *and keep agreements and promises especially reliably now*. (S3 File)

There are also materials that focus on specific holidays like Halloween, Thanksgiving, Christmas, and Chinese New Year. New ways of spending time were proposed as the traditional ways of celebrating calendar events were no longer considered safe. Meetings with family and friends, trips to relatives, and visiting grandparents were risky, and everything needed to be organized in accordance with sanitary rules, i.e. primarily in isolation and at a distance (S3 File).

Cultural specificity is particularly evident here. Educational materials prepared by governmental organizations refer to holidays that are significant in that specific culture. However, there is a paucity of materials that address minority cultures in individual countries. While there is generally a balance in terms of gender and cultural diversity in the general context of holidays, there was no reference to holidays celebrated by minority groups, such as Eid for Muslims in Germany.

Despite the fact that, almost in parallel to the struggles caused by the pandemic, many national and international institutions as well as the media were reporting on the threats of the infodemic, relatively few educational materials addressed these phenomena. Some aspects of misinformation were briefly presented in 26 materials, mainly in those designed for parents and teachers. They recommended staying informed, using reliable sources, guiding children through the available information, and informing children about the need of filtering news, as well as controlling screen time and the media sources used. For example:

*If you can’t answer their questions*, *don’t guess*. *Use it as an opportunity to explore the answers together*. *Websites of international organizations like UNICEF and the World Health Organization are great sources of information*. *Explain that some information online isn’t accurate*, *and that it’s best to trust the experts*. (S3 File).

Some of the materials instructed parents and teachers on how to talk with children about the pandemic situation and which information to share with them. However, it was only very rarely that the materials addressed the need of developing children’s critical thinking and health literacy. One of the WHO documents targeted at teachers mentioned:

*Media literacy lessons can empower pupils to be critical thinkers and makers*, *effective communicators and active citizens*. (S3 File)

While the materials designed for children seem to ignore these issues altogether, those targeting adolescents only rarely recognize the confusions and stress that widespread misinformation can cause in young people. One of the Chinese videos that provided guidance on adapting to the psychological challenges of the pandemic warns an adolescent audience that some sources of information are credible (the national media, government agencies, and the website of the Health Ministry) while others are unreliable (social media, internet forums, and private messages).

The time of the pandemic has proved to be a difficult period, and is identified as such in the educational materials for children and adolescents. Much emphasis is placed on issues related to mental and social health. In all of the countries analyzed, attention is paid to the need to care for mental well-being. Parents and guardians also receive advice on how to care for their charges and how to better cope with difficulties. Indeed, difficulties are presented as challenges that can be dealt with rather than as insuperable problems. The virus is presented as a threat, but we do not see too much exaggeration or exposure to the threat. The dominant attitude towards the dissolution of the will and dealing with the fallout leaves no room for asking "why", though this is a key question in critical thinking. The educational materials are instructional rather than informative: they provide solutions, rather than seeking to educate their audience about viruses or epidemiology. The educational strategy adopted does not, however, enable the development of critical thinking, and thus is not effective in the fight against the infodemic.

## 4. Discussion

All of the educational materials offer simple instructions about desirable behaviour in the pandemic. The materials produced in different countries prioritize each of the messages differently. The key messages regard the importance of washing hands, maintaining social distance, and wearing masks, and were often complemented with instructions on how to perform each task effectively. Short, informative instruction are probably the consequences of a rapid response. The state of the pandemic itself is presented differently across the materials analyzed. However, the dominant narrative is that it is a stressful situation for all people, including children and adolescents, and is one that can cause emotional and social crises. Therefore, in the educational materials designed for these two audiences, in addition to tips on washing hands and maintaining social distance, there are also tips on caring for one’s mental and social well-being.

The educational materials can be considered as instructional, with numerous recommendations and precise instructions on how to behave. However, referring to the aforementioned concept of Zarcadoolas et al. [14] which points to the four dimensions of HL, it is clear that not all of them are developed. While pupils do indeed learn to understand the new social situation and the cultural contexts of the pandemic (cultural literacy), they learn relatively little that would contribute to their science literacy that helps to interpret epidemiological and medical data, and the civic literacy that is responsible for critical media reception. While such a narrative—directive, instructive, emphasizing the need to take specific actions (or refrain from doing so)—is helpful in the initial phase of the pandemic as a new phenomenon we have all faced, we question whether it is sufficient.

The material only briefly mentions the threat of unverified information sources. Very general information is provided, though this is addressed to adults rather than children and adolescents. Media all over the world [according to Luhmann’s concept, 21] have built their message on numbers. Every day in the pandemic, we receive a report about new cases, the numbers of deaths and other statistics. This message based on numbers has built fear, and perhaps also stimulated the feeling of individual responsibility. These same numbers and statistics, however, are susceptible to manipulation and unauthorized use. This situation, which is characteristic the current and probably also future current and probably also future stages of the pandemic and probably of its later stages, already requires skills beyond the simple knowledge of measures to prevent infection or to mitigate the consequences of lockdown and social isolation. It requires the development in children and young people of the ability to analyze facts and opinions, to form an opinion or to base one on scientific grounds, and thus to build critical thinking skills about health, and consequently to develop health literacy.

The concept of critical thinking is marginal in the materials analyzed. It occurs most often in the context of the suggestion to allow children to ask questions and to respond honestly. Only in a small sample of materials posted on social media, do the authors encourage their audience to click on the supplied link to get more information or to discuss the problem. However, the chat threads that appear beneath such posts do not generally provoke a reaction. American organizations encourage parents to limit the access of children and adolescents to the media. Several documents also point out that some information in the media can be misleading and / or untrue. However, we in the materials included in the analysis we did not find instructions on how to verify information or how to deal with fake news.

To our knowledge, this is the first study to analyze materials educating children and adolescents on the threat of the pandemic from various cultural contexts in reference to health literacy and critical thinking. One of the limitations of our analysis is the limited number of countries from which such educational materials were retrieved. However, those countries that were studied are based on three different continents—North America, Asia and Europe (Western, Southern and Eastern)—use different languages, experienced the pandemic in different points of time and at diverse intensity levels, have different healthcare systems and different educational capitals of their national educational systems, and therefore constitute a heterogeneous sample. Moreover, we also included educational materials from international organizations, with these materials being available to all countries in the world. In addition, different information strategies were used, with some countries disseminating international materials and recommended materials from NGOs, and offered redirections from government websites to the materials they published. In the case of China, however, only those materials prepared and approved by the state authorities were admissible.

Another limitation is that our analysis is not exhaustive, as it covers only a choice of educational materials in a given country, restricted to the official governmental sources or those recommended by that governmental source, while there were many bottom-up initiatives from nongovernmental organizations and individual persons that have not been included in this study. However, the analysis presented in this paper allowed for a sharper focus on the official materials that were prepared, issued, or officially endorsed in a given country. In our study, we have also focused solely on the content of the materials and have not analyzed their reliability nor readability. It could be an interesting area for further research.

## 5. Conclusions

Beyond the pandemic, the WHO has clearly communicated their stance on the infodemic and the importance of fighting misinformation. Numerous organizations have engaged in the fight against the infodemic, including social media platforms such as Facebook. This notion however is not reflected in the materials analyzed directed at children or youth(even those prepared by the WHO). While HL is a resource that enables an individual to act upoin health information, the analyzed educational content conveys very simple instructions outlining behavioural changes crucial in preventing viral infection or promoting cross-generational solidarity. Most of the materials fail to question the reliability of the information available, and lack encouragements to ask questions–a vital for fighting the infodemic.

Based on the material analyzed from three continents, we have established that, regardless of the pandemic context, none of the national nor international actors have managed to publish a coherent set of didactic materials that would truly promote health literacy. One explanation may be the need to respond quickly to the crisis. The content of the educational materials developed as the scientific understanding of the coronavirus as well as national or international strategies towards COVID-19 evolved. At the beginning, the mechanisms of the virus and the level of contagiousness were not fully known, hence the predominant aspect presented in the educational materials reinforced the basic countermeasures, i.e. promoting hygiene, disinfection and distancing. In the next step, information on psychological support and materials informing how to cope with lockdown and forced restriction of all outside activities outside were taken care of. Considering the need of an instant response, we appreciate the content of the initial materials at the very beginning of the pandemic. However, they did not evolve into resources strengthening health literacy nor critical thinking about health. Given the following months of restriction fatigue and rapidly growing distrust of vaccination, in our view it has become a missed opportunity.

In that respect we see that some of the actors responsible for creating COVID-19 educational materials have attempted to signal their authority and expertise in the medical field. This strategy of persuasion could provide a key element in a successful educational message, one that stands in the face of the era of the infodemic and the abundance of unreliable sources, namely, the recipient’s trust. Considering the epidemiological predictions of subsequent global health crises in the future [22], the development of HL among the young generation appears to be a common goal for all countries. As we have seen in the materials produced to educate people about COVID-19, which used the image of the ancient Chinese philosopher Confucius and a symbol of British pop culture, the expertise in health education should focus on enhancing the individuals’ ability to take informed decisions on health all over the world. Providing informative and adapted to recipients’ needs educational materials on health is crucial also in the context of vaccine hesitancy observed to have increased especially during the initial phase of the pandemic. Developing health literacy in children and the youth might support the process of decision-making in regard to important health issues as the tendency to delay acceptance or refuse to vaccinate, which was found to be impacted by contextual factors, among others younger age and a lower level of education, vaccine related determinants, such as for example belief that vaccines are not safe or effective, and group/individual factors, among others less trust in science and healthcare system [23].

### 5.1. Recommendations for practice

Based on the conducted analysis of the educational materials from five countries and international organizations we developed three recommendations regarding the process of development of health educational resources. Firstly, health issues should be embedded in the school curriculum, based on evidence and trustworthy guideline recommendations. In emergency situations, such as a pandemic, instructive materials could help promoting new behaviors but might be not enough to shape healthy attitudes. Health themes should be discussed interdisciplinary and critical thinking competences should be promoted along this process using adequate methodology.

Secondly, we recommend adapting linguistically and visually the message on health to the cognitive, psychological and social needs of the targeted audience. Good examples of materials adjusted to children’s perception are the PAHO resources using cartoon characters to create the narration on COVID-19 or posts published by the American portal Engage Youth 4 Change applying numerous visual communication strategies typical for social media to reach young people’s attention. We recommend that educative materials are created in accordance with the postulates of equity in education. It is crucial to address the socio-cultural diversity of the potential material users as well as special needs resulting from disabilities. Children and teenagers’ participation in the material development and validation process could resolve the challenge of materials readability and accessibility.

Finally, the pandemic revealed how crucial for children’ and teenagers’ wellbeing are knowledge and competences in the field of mental health. Topics regarding individual resources, stimulating emotional intelligence and strengthening resilience were crucial for adaptation to pandemic challenges however their usefulness remains. Therefore, we strongly recommend that mental health is placed at the heart of health education curricula.

## Supporting information

S1 File Comparison of epidemiological data in countries included in the analysis. (DOCX)

S2 File Code book: Name of the main codes and subcodes (number of coded segments). (DOCX)

S3 File Detailed information on the materials quoted in the article. (DOCX)
