# Supplementary material for: When Peppa Pig and Confucius meet, joining forces on the battlefield of health literacy–a qualitative analysis of COVID-19 educational materials for children and adolescents from China, the USA, and Europe
Source: PLoS One. 2022 Dec 6;17(12):e0278554. doi: 10.1371/journal.pone.0278554 (PMC9725119; doi:10.1371/journal.pone.0278554)

**Supplementary material, Part 1**

**Comparison of epidemiological data in countries included in the analysis**

Figure 1. Number of confirmed cases per 100,000 people, as of August 18th 2021 (<https://covid19.who.int>)


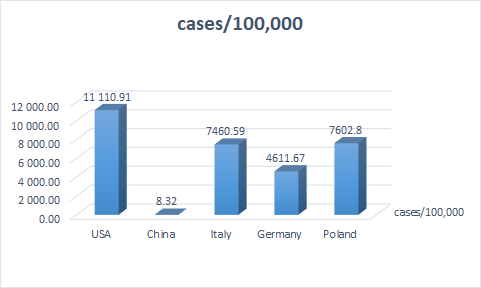


Figure 2. Number of deaths due to COVID-19 per 100,000 people, as of August 18th 2021

(<https://covid19.who.int>)


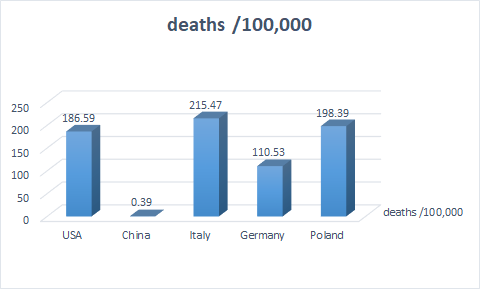


Figure 3. Case fatality rate (%) for COVID-19 , as of August 18th 2021

(<https://covid19.who.int>)


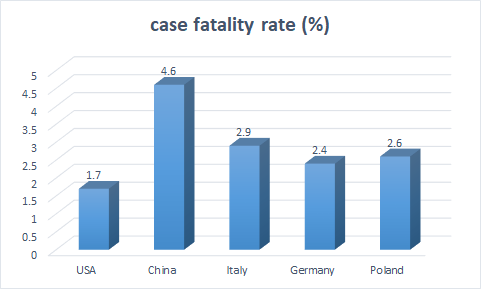


Figure 4. Full COVID-19 vaccination update (% of total population) of August 19th 2021

(<https://vaccinetracker.ecdc.europa.eu/public/extensions/COVID-19/vaccine-tracker.html#uptake-tab> <https://ourworldindata.org/covid-vaccinations>)


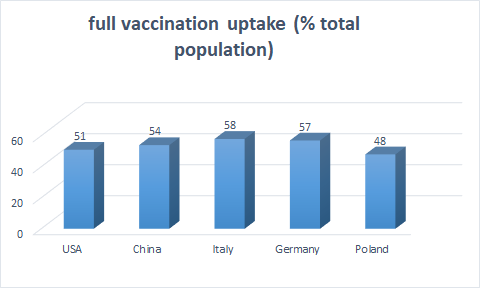

Supplement: S1 File — (DOCX) [file pone.0278554.s001.docx]
