# Supplementary material for: When Peppa Pig and Confucius meet, joining forces on the battlefield of health literacy–a qualitative analysis of COVID-19 educational materials for children and adolescents from China, the USA, and Europe
Source: PLoS One. 2022 Dec 6;17(12):e0278554. doi: 10.1371/journal.pone.0278554 (PMC9725119; doi:10.1371/journal.pone.0278554)
Supplement: S2 File — (DOCX) [file pone.0278554.s002.docx]

**Supplementary materials, Part 2**

**Code book: name of the main codes and subcodes (number of coded segments)**

***content of the message*** *2218*

*general information about viruses 44*

*general information on SARS-COV-2 70*

*COVID-19 symptoms 76*

*COVID-19 treatment 20*

*spreading paths 103*

*mortality (numbers) 5*

*number of cases 6*

*who gets sick 42*

*wearing masks / face mask 128*

*washing hands 240*

*disinfection 69*

*avoiding contact 95*

*keeping distance 105*

*prophylaxis - other recommendations 234*

*quarantine 26*

*Self-isolation 37*

*lockdown 23*

*vaccines 25*

*physical health 77*

*mental health 251*

*social health 160*

*daily challenges in a pandemic 133*

*social problems in a pandemic 31*

*distance learning 33*

*read more 117*

*other 67*

***type of knowledge***

*lay knowledge 76*

*general expert 95*

*medical expert 78*

***who is the hero***

*Virus 43*

*a specific cultural figure 27*

*undefined teenager 15*

*undefined adult 26*

*unspecified child 58*

*unspecified parent 20*

*unspecified teacher 5*

*psychologist 6*

*nurse / nurse 1*

*doctor / doctor 15*

*"alternative medicine specialist" 0*

*politician, celebrity 0*

*Other expert ( e.g. scientist, researcher, statistician) 17*

*other 23*

***How the content is delivered***

*"advising" 140*

*positive example 63*

*factual 73*

*scaring / blackmailing 14*

*instruction 217*

*encouraging action 112*

*what not to do 66*

***Language applied***

*scientific 12*

*colloquial 223*

***Virus names used***

*coronovirus 105*

*covid-19 164*

*COVID-19 16*

*SARS-CoV_2 10*

*othera 80*

***metaphors***

*virus as a monster 34*

*fighting / wars 57*

*cooperation 40*

*other 2*

***emotions***

*negative 18*

*positive 65*

*ambivalent 7*

*none 106*

*other 5*

***critical-thinking details***

*infodemia 39*

*reference to fakenews 20*

*giving sources 20*

*possibility of commenting 20*

*possibility to ask questions 13*

***visual dimmension***

*light icon 0*

*prohibition icon 28*

*food icon 39*

*handkerchief icon 46*

*water tap 83*

*face mask icons 99*

*virus icon 174*

*children icon 163*

*iconic youth 43*

*family icon 34*

*adult + child icon 84*

*distance icon 11*

*other icons 284*

***audio dimmension***

*music from a horror / drama 6*

*unidentified music theme 9*

*famous music theme 5*

*music that encourages action 29*

*music builds a positive mood 82*

***cultural specificity 105***

***gender of the figures presented***

*Only female 93*

*Only male 126*

*mixed 110*

*none 66*
