# Supplementary material for: When Peppa Pig and Confucius meet, joining forces on the battlefield of health literacy–a qualitative analysis of COVID-19 educational materials for children and adolescents from China, the USA, and Europe
Source: PLoS One. 2022 Dec 6;17(12):e0278554. doi: 10.1371/journal.pone.0278554 (PMC9725119; doi:10.1371/journal.pone.0278554)
Supplement: S3 File — (DOCX) [file pone.0278554.s003.docx]

| **Supplementary materials, Part 2**  **Detailed information on the materials quoted in the article.** | | | |
| --- | --- | --- | --- |
| **Data excerpt no** | **Country/ publishing actor** | **Description of the educational material** | **Link to the source** |
| 1 | China | Picture one page 14. “Doctors nurses, scientific research experts, people's police, journalists, infrastructure workers. They are all in their respective front positions, contributing their own strength to the protection of health! Virus,virus I am not afraid!” (Translated from Chinese by Author)  “医生护士，科研专家，人民警察，新闻记者，基建工人他们都在各自的岗位上，为守护健康贡献着自己的力量. 病毒病毒，我不怕” | https://lifetreebooks.org.cn/pc/index/read.html?id=100 , p.14 |
| 2 | International organization PAHO | Virus tends to be pictured in various educational materials as wearing a crown. Examples from different countries:   1. “Wicked virus and powerful children” - picture book for children 2. “How the virus gets his name” - video for children about virus 3. “Storia di un coronovirus” - picuture book for Italian children about coronovirus | 1.  <https://www.paho.org/en/documents/wicked-virus-and-powerful-children>  2.  <https://www.youtube.com/watch?v=KpXZkChOXwI&list=PLvrp9iOILTQaJa78zFQ0QgvShQ2HEwHxP&index=99>  3. <https://www.policlinico.mi.it/uploads/fom/attachments/pagine/pagine_m/78/files/allegati/546/storia_di_un_coronavirus_-_alfabetico_-_secondo_finale.pdf> |
| 3 | China | A picture illustrating little Chinese girl presenting how children can fight with the virus. They need follow official instructions and respect their caregivers  “早睡早起多活动，读书画画做游戏。我是健康好宝宝，病毒来了我不怕！”  “Go to bed early and get up early to do more physical exercise and activities like reading, drawing and playing games. I am a healthy baby, I am not afraid of the virus!” (Translated from Chinese by Author) | <https://www.sohu.com/a/370143090_120151622>, picture 12 |
| 4. | China | In the picturebook entitled “Novel coronavirus. Children’ Epidemic Prevention Picture Boo”  a novel coronavirus is called ‘little monster’. The book depicts the beginning of the epidemy, its source and symptomps of the infection | <https://www.meipian.cn/2rcbk17o> |
| 5 | Germany | A picture from the comic strip “Agenten im Einsatz” (eng. Agents in action) shows an army of viruses represented as monsters, carrying a banner with the inscription "Mission - Human" and the comment: evil can hide in our organisms. | https://www.bzga.de/infomaterialien/impfungen-und-persoenlicher-infektionsschutz/hygiene/agenten-im-einsatz/ |
| 6 | International organization | The book “Wicked virus and powerful children” illustrates the virus as an ugly creation with a green body, one eye, and a crown. Beto, Bial Biel are small playful children who enjoy imagining they are heroes. When faced with the information about the threat of coronavirus, they come up with effective solutions, such as washing hands, staying home and doing arts, to cope with adversaries of the pandemic. | <https://www.paho.org/en/documents/wicked-virus-and-powerful-children> |
| 7 | International organization | “My hero is you: How kids can fight COVID-19" is an illustrated story about Sara who learns from her mum, a scientist, how to fight coronavirus. In her dreams, Sara travels with a Dragon Ario all over the world and visits other children. They encourage them to stay home, wash hands, keep distance, follow coughing etiquette. The story covers also the notions of missing grandparents, being patient and understanding towards each other, showing care with words rather than with a hug, and care for each other. | https://www.unicef.org/coronavirus/my-hero-you |
| 8 | USA | “Campers, get ready to stop COVID-19 in its tracks" - a video dedicated to children and adolescents planning to go to summer camp. The symbols of pandemic restrictions are visuals in shape of scouts’ badges and conforming to them presented as scouts’ asset. The titles given to those following the restrictions are formulated using terms associated with fighting an enemy, courage and success. | https://www.youtube.com/watch?v=fqpQhuWYOtc&list=PLvrp9iOILTQaJa78zFQ0QgvShQ2HEwHxP&index=54 |
| 9 | China | “Together we fight against epidemic. Do a good job, take precautions. Do not believe in information from untrusted sources., this quote appears on the end of the video Confucius explain to his apprentice the pandemic circumstances. E.g. He teaches him how to wash hands properly and wear a face mask. The video was published on youku.com platform for general audience. | https://v.youku.com/v_show/id_XNDU0OTk5NjE5Mg==, 1:48 from the beginning of the movie |
| 10 | China | Quote from picturebook which underline how human were infected with the new coronavirus.  “找到了他的收货人，一手交钱一手交货。但是他们却不知道其中有一只小动物身上携带了」种未知病毒。这种病毒是一种微小的非细胞生物，你的眼睛看不到它，但是在放大镜下，科学家发现这个新病毒的形状好像皇冠一样，这种皇冠状病毒一直寄生在野生动物身上”  “The hunter found the man who was responsible for taking the delivery of the goods. He gave him the hunted animals and got some money. But they did not know that one of the small animals carried an unknown virus. This virus is a tiny non-cellular organism which the human eye cannot see. But it was discovered by scientists under an electron microscope and its spikes resembled a crown. The virus with crown-like spikes on its surface has been living in wild animals and feeding from them” (Translated from Chinese by Author) | http://zj.people.com.cn/n2/2020/0129/c186327-33749778.html p.4 |
| 11 | USA | Instagram story *How to slow the spread of COVID-19* prepared by CDC with lots of iconic pictures.  *“Do them all to best protect yourself, your family, your friends, and those more likely to get very sick from* [*#COVID19”*](https://www.instagram.com/explore/tags/covid19/)*.* | https://www.instagram.com/p/CMC_jcxgy8c/?utm_source=ig_web_copy_link |
| 12 | Italy | The story about a girl who is dealing with her pain after losing her grandpa. The story describes that a letter, that will be delivered by Tooth Fairy, to dead grandpa can sooth the grief. | https://www.policlinico.mi.it/uploads/fom/attachments/pagine/pagine_m/78/files/allegati/586/storia_di_un_coronavirus_-_una_letterina_per_te_nonno.pdf |
| 13 | China | Posters with very detailed instruction about washing hands  “Wash your hands with soap, wash your hands with running water.  You must use running water and soap or hand-sanitizer. Time of washing hands have to be longer than 20 seconds. Hand washing instructions in 7 steps.   1. Face your palms together with fingers close to each other and rub them. 2. Rub your palm against the back of the other hand. Change hands. 3. Face your palms to each other, cross your hands, rub your fingers joints. 4. Interlock your half-clenched fist and rub the back of them against your palms. 5. Hold the thump in one hand, rub and rotate. 6. Put the five fingertips together, rub with the other palm and rotate. Change hands 7. Rub your wrists and arms. Change your hands.” | http://www.moe.gov.cn/jyb_xwfb/s7600/202004/t20200414_443274.html |
| 14 | Poland | A poster dedicated to children presents them what to do (washing hands 30 seconds, before eating, how to follow sneezing and coughing etiquette) and explains why washing hands is so important. The title is:  “Avoid touching eyes, nose and mouth. Hands touch many surfaces which can be contaminated with the virus" (translated form Polish by Author) | <https://www.gov.pl/web/edukacja-i-nauka/materialy-informacyjne-dla-szkol-przedszkoli-i-placowek-oswiatowych-dotyczace-profilaktyki-zdrowotnej> |
| 15 | Italy | *Video for kids showing how to wash hands. Two hands are washing themself and singing:*  *Le mani, le mani, dobbiam lavare le mani.*  *Con acqua e sapone quaranta secondi le mani, le mani.*  *Un palmo, poi l’altro di qua e di la, incrocia le dita non ti fermar, destra, sinistra, su e giù, continua ancora un po’.*  *Un pugno, poi l’altro fai così, il pollice stringi qui e li.*  *Dei cerchi ancora dobbiamo far prima di terminar.*  *Le mani, le mani, dobbiam lavar le mani.*  *Risciacqua, asciuga e batti pulite le mani, le mani”.*  *Hands, hands, we have to wash our hands.*  *With soap and water forty seconds hands, hands*  *One palm, then the other on both sides, cross your fingers do not stop, right, left, up and down, continue a little longer.*  *One fist, then the other do so, the thumb squeeze here and there.*  *We still have to do some circles before finishing.*  *Hands, hands, we must wash our hands.*  *Rinse, dry and clap your hands clean, hands (Translated from Italian by Author)* | https://it-it.facebook.com/CroceRossaDesio/videos/dobbiam-lavare-le-manibellissima-canzoncina-da-insegnare-ai-nostri-bimbi-unoccas/208622866876499/ |
| 16 | International organization | A video by WHO and PAHO reminds children to wash hands. Peppa Pig and friends are presented while washing hands. The bright colours, singing shows that washing hands is fun. Also Elmo and other characters from Sesame Streat shows how to have fun washing hands and how to do it properly  More examples with Baby Shark are on the PAHO webpage dedicated to parents | <https://www.paho.org/en/covid-19-resources-parents-and-children> |
| 17 | China | Instruction on how to wear face masks properly also showing examples of masks that provide the appropriate level of protection and the ones which do not provide such guarantee | https://lifetreebooks.org.cn/pc/index/read.html?id=48 , p.32 |
| 18 | Germany | Excerpt from a Youtube video “Alltagsmasken für Kinder erklärt” (Everyday masks for children explained) made for children by the Bundesministerium für Gesundheit (Federal Ministry of Health). A girl tells other children why masks must be worn, but also how important it is to wear them correctly. A boy who has one on incorrectly is attacked by viruses and frightened. The girl shows him in a few steps how to put on and take off the mask correctly, and not to forget to wash your hands afterwards. | https://www.youtube.com/watch?v=zEVZ2JjtZ4k |
| 19 | *USA* | *Instagram post about washing cloth mask.*  *How to wash your cloth masks. ���� @cdcgov*  *DYK? Cloth masks should be washed after each use. It is important to always wear and remove your mask correctly. Wash your hands after wearing, handling, or touching your mask. Masks, when combined with other every day steps like staying 6 feet apart and washing your hands often, help slow the spread of #COVID19. Learn more:* [*http://bit.ly/CDC-WashMasks*](http://bit.ly/CDC-WashMasks)*. #PublicHealth #Health #CDC #WearAMask* | https://www.instagram.com/p/CGko2rvDl8E/?utm_source=ig_web_copy_link |
| 20 | Germany | Excerpt from a Youtube video “Zehn Verhaltenstipps von Max und Flocke in Zeiten von Corona“ (Ten tips for behaviour from Max and Flocke in times of Corona) prepared for children by the Bundesamt für Bevölkerungsschutz und Katastrophenhilfe (Federal Office for Civil Protection and Disaster Assistance) with tips on how to cope during a pandemic. The narrator, a roughly ten-year-old boy Max, owner of the dog Flocke, tells the other children what life is like now and what recommendations to follow (masks, distance). He also points out that although you can't visit relatives, it's a good idea to connect with them online (e.g. grandparents), and to meet friends outside and combine socialising with exercise and sport. | https://www.youtube.com/watch?v=2vTjYGqGCpQ |
| 21 | USA | Article in the CDC webpage *Take care of your body. Take deep breaths, stretch, or meditate. Try to eat healthy, well-balanced meals. Exercise regularly. Get plenty of sleep. Avoid excessive alcohol, tobacco, and substance use. Continue with routine preventive measures (such as vaccinations, cancer screenings, etc.) as recommended by your healthcare provider.*  *Get vaccinated with a COVID-19 vaccine when available* | https://www.cdc.gov/mentalhealth/stress-coping/cope-with-stress/index.html?CDC_AA_refVal=https%3A%2F%2Fwww.cdc.gov%2Fcoronavirus%2F2019-ncov%2Fdaily-life-coping%2Fmanaging-stress-anxiety.html |
| 22 | Germany | *Quoting from the website of the NGO Kinderhilfswerk, to which the government website of Bundeszentrale für gesundheitliche Aufklärung (Federal Centre for Health Education) redirects. This page is intended for children aged 8-13, the quoted passage is an attempt to answer in a child-appropriate way the most common questions related to the pandemic.*  “Warum dauert es so lange mit dem Impfstoff?  Damit Menschen vor dem Coronavirus geschützt werden können, haben Wissenschaftlerinnen und Wissenschaftler einen Impfstoff entwickelt. Eigentlich dauert es ziemlich lange, einen Impfstoff zu entwickeln. Es müssen Informationen über das Virus gesammelt werden. Gleichzeitig soll der Impfstoff sicher sein und muss an sehr vielen Testpersonen untersucht werden. Besonders wichtig ist es dabei, Nebenwirkungen auszuschließen.” | *https://www.kindersache.de/bereiche/wissen/natur-und-mensch/die-wichtigsten-fragen-zum-coronavirus* |
| 23 | International Organization | *UNICEF prepared webpage about how do covid vaccine work for several audiences.* In a short video, a doctor talks with a little boy,a parent, PhD student and an expert and explains what the vaccine is all about and how it works | https://www.youtube.com/watch?v=n5CLKOnfBuM&t=13s |
| 24 | Germany | a) An animation prepared for youth and adults by *Bundeszentrale für gesundheitliche Aufklärung (Federal Centre for Health Education)* showing how to contract coronavirus (and other droplet-transmitted viruses).  b) An exceprt from Youtube video “Alltagsmasken für Kinder erklärt” (Everyday masks for children explained) made for children by the Bundesministerium für Gesundheit (Federal Ministry of Health) | a) https://www.infektionsschutz.de/fileadmin/impfen-info.de/Videos/Troepfcheninfektion/Animierte_Infografik_Uebertragung_Luft_video_v0.0_1.mp4  b) https://www.youtube.com/watch?v=zEVZ2JjtZ4k |
| 25 | Poland | A brief article describes the emotional consequences that might potentially result from the pandemic and promotes free of charge service offering video-call consultations with psychologists. | <https://www.drabina.org/zapytaj/index.htm> |
| 26 | USA | An Instagram post encourages to avoid too stressful situation and setting boundaries to protect ones’ own health. | https://www.instagram.com/p/CIBqyWZFqGM/?utm_source=ig_web_copy_link |
| 27 | International Organization | Elmo’s mum from Sesame Street shares her struggle with parents and advice talking about emotions with children | https://www.unicef.org/parenting/care/supporting-elmo-covid-19 |
| 28 | USA | Internet page with tips how to set up *routines and schedules at home, and keep your child focused on these.* | https://engage.youth.gov/resources/five-tips-tackling-changes-your-schoolwork-routine |
| 29 | USA | An article promoting physical activity and healthy diet dedicated to teenager. Lots of practical information how to be fit and health | <https://engage.youth.gov/resources/take-charge-your-health> |
| 30 | Italy | The instruction for adolescents between 12 and 17 years old that gives an example of everyday schedule of physical activities connected with helping in the routine housework | https://www.policlinico.mi.it/coronavirus-spiegato-a-bambini-e-adolescenti |
| 31 | Poland | A list of contacts for children and adolescents needing a psychological support due to emotional problems caused by the pandemic | <https://bip.brpo.gov.pl/pl/content/pomoc-psychologiczna-pandemi%C4%85-covid-19-faq> |
| 32 | Germany | Material prepared for parents by the Bundesamt für Bevölkerungsschutz und Katastrophenhilfe (Federal Office for Civil Protection and Disaster Assistance). It provides tips on how to care for and support children during a pandemic (addresses physical, mental and social challenges related to disease risk, lockdown and restrictions. | <https://www.bbk.bund.de/SharedDocs/Downloads/DE/Mediathek/Publikationen/Buergerinformationen/A4-Buergerinfo/covid-19-tipp-eltern.pdf?__blob=publicationFile&v=2> |
| 33 | USA | An Instagram graphic providing tips for celebrating Thanksgiving according to the pandemic restrictions with the background stylized as a festive postcard  “Attending a Thanksgiving gathering this year? Take steps to make #Thanksgiving safer.  #WearAMask, stay at least 6 feet apart, and wash your hands often.  Choose outdoor or well-ventilated spaces. Check out @cdcgov for more tips | https://www.instagram.com/p/CH8TgqLrZmR/?utm_source=ig_web_copy_link |
| 34 | International Organization | *If you can’t answer their questions, don’t guess. Use it as an opportunity to explore the answers together. Websites of international organizations like UNICEF and the World Health Organization are great sources of information. Explain that some information online isn’t accurate, and that it’s best to trust the experts.* | <https://www.unicef.org/coronavirus/how-talk-your-child-about-coronavirus-covid-19> |
| 35 | International Organization | A video presented by a UNICEF Senior Producer, sharing UNICEF experts’ recommendations on how to talk with children about the pandemic. | <https://www.unicef.org/coronavirus/how-talk-your-child-about-coronavirus-covid-19> |
| 36 | International Organization | A document by WHO entitled *‘*Key Messages and Actions for COVID-19 Prevention and Control in Schools’ offers set of recommendations how to organize schools in the pandemics. | https://www.who.int/publications/m/item/key-messages-and-actions-for-covid-19-prevention-and-control-in-schools |
